# Antioxidant and cytotoxic potentials of the methanolic extract of *Teucrium persicum* Boiss. in A-375 melanoma cells

**DOI:** 10.22038/AJP.2021.19308

**Published:** 2022

**Authors:** Anahita Naeimi, Majid Tafrihi, Maryam Mohadjerani

**Affiliations:** *Department of Molecular and Cell Biology, Faculty of Basic Sciences, University of Mazandaran, Babolsar, Iran*

**Keywords:** Teucrium persicum, A-375 cells, Antioxidant potential, Cytotoxicity, Caspase 3/7, Genotoxicity

## Abstract

**Objective:**

*Teucrium persicum* is an Iranian endemic plant used in Iranian traditional medicine.

**Materials and Methods:**

The total phenolic and total flavonoid contents, and antioxidant potential of the methanolic extract of *T. persicum* were determined. The MTT test was used to evaluate the inhibitory effect of the extract on the viability of A-375 cells. The clonogenic, micronucleus formation, and acridine orange/ethidium bromide staining methods were used to evaluate the survival and proliferation of A-375 cells. Apoptosis was evaluated by using DNA fragmentation assay and measuring the activity of caspase 3/7. To study the effect of the extract on the migration of A-375 cells, the *in vitro* wound-healing (scratch) assay was employed.

**Results:**

The average total phenolic and flavonoid contents and antioxidant properties of the extract were 6.97±0.011 mg Ellagic acid (EGA)/g, 46.83±0.0019 mg of the ethoxyquin (1,2-dihydro-6-ethoxy-2,2,4-trimethylquinoline; EQ)/g of dried extract, and 10±0.002 μg/ml, respectively. The IC_50_ value of the *T. persicum* methanolic extract was 13 μg/ml for 48 hr. The DNA fragmentation pattern and the activity of caspase3/7 suggested that the reduction of the cell viability may be due to apoptosis induction. Microscopic observations showed nuclear condensation, a considerable increase in micronuclei formation, and inhibition of the colony formation in A-375 cells treated with 7 μg/ml to 15 μg/ml of the extract. Wound-healing assay supported the anti-migration activity of the extract.

**Conclusion:**

*T. persicum* has significant antioxidant and cytotoxic properties. Surely, more detailed molecular and biochemical studies are needed to find the mechanism(s) behind these effects.

## Introduction

Melanoma is a malignant skin tumor arising from the malignant transformation of the melanocytes (Das et al., 2016 ▶). Several factors affect susceptibility to melanoma, including genetic and environmental factors (Chinembiri et al., 2014 ▶).

The use of plant-derived phytochemicals as therapeutic agents has a long history. Several reports show that some plant-derived compounds including vinblastine, vincristine, doxorubicin, topotecan, etc. are used clinically to treat various human cancers (Cragg and Newman, 2005 ▶; Hue et al., 2012 ▶).

*Teucrium *is a genus that belongs to the Lamiaceae (Labiatae) family (Abdolghaffari et al., 2010 ▶). This genus includes more than 300 species that are widely distributed in the world (Amirghofran et al., 2010 ▶). It has been reported that 12 species are growing in Iran. *Teucrium melissoides *Boiss et. Hausskn, *T. macrum Boiss et. Hausskn*, and *T. persicum *Boiss are known as Iranian endemic species (Masoudi et al., 2009 ▶). *Teucrium* species are traditionally used to treat various human diseases (Morteza-Semnani et al., 2007 ▶; Al-Hamwi et al., 2021 ▶). Also, some *Teucrium* species show anti-microbial, antioxidant, anti-nociceptive, anti-allergic, and anti-cancer potentials (Rajabalian, 2008 ▶; Movahedi et al., 2014 ▶; Rizvi et al., 2019 ▶).

*T. persicum* grows in Fars province and is used in traditional medicine to alleviate headaches and ventral pains (Monsef-Esfahani et al., 2010 ▶). Several investigations have identified the chemical profile of the essential oils of *T. persicum *(Javidnia et al., 2007 ▶; Miri et al., 2012 ▶). A few *in vivo* studies assessed biological effects of *T. persicum* (Rasekh et al., 2005 ▶; Miri et al., 2015 ▶), however, there is no report about the cytotoxic and genotoxic effects of *T. persicum* on the A-375 cell line as a cancer model. Therefore, in this study, A-375 cells have been used to investigate the cytotoxic and genotoxic potentials of *T. persicum*. 

## Materials and Methods

**Methanolic extract preparation**

*Teucrium persicum* was collected from the south of Iran and their identity was confirmed by Dr. Alireza Naqinezhad from the Department of Biology, Faculty of Sciences, University of Mazandaran (9008-HUMZ). Then, 50 g of the aerial parts of *T. persicum* was powdered and then soaked in 100 ml of pure methanol and shaken for 48 hr. The evaporation process of the supernatant was performed by a rotary evaporator (Heidolph, Germany) at 37ºC. Freeze-drying of the extract was performed by using a freeze-dryer (Christ, Germany). Finally, 50 μg/ml concentration of the extract was prepared as a stock and kept at -20°C for treatments.

**Determination of total phenolic contents**

The total phenolic contents (TPC) of *T. persicum* methanolic extract were assessed according to Singleton and Rossi using the Folin-Ciocalteu method (Singleton and Rossi, 1965 ▶). Gallic acid was used as a standard, and the TPC was expressed as milligram of gallic acid equivalents/gram of the extract on a dry basis.

**Determination of total flavonoid contents**

The total flavonoid contents (TFC) of *T. persicum* methanolic extract were assayed by using the aluminum chloride colorimetric method. Briefly, different concentrations (75-125 µg/ml) of the extract were separately mixed with 1.5 ml of ddH_2_O and 0.075 ml of 5% NaNO_2 _for 5 min. Then, 0.15 ml of 10% AlCl_3_ was added. After 6 min, 0.5 ml of 1M NaOH, and 2.8 ml of distilled water were added and kept at room temperature for 30 min. The absorbance of the mixture was then measured at 510 nm by an ELISA reader (BioTek, USA). Quercetin was used as a standard reference, and the TFC was expressed as milligram of quercetin equivalents per gram of the extract on a dry basis. All assays were accomplished in triplicates.

**Evaluating the 2, 2-diphenyl-1-picrylhydrazyl (DPPH) radical scavenging potential**

One milliliter of 0.1 mM solution of DPPH radical in methanol was added to different concentrations (20, 50, and 100 µg/ml) of *T. persicum* methanolic extract. The mixture was vortexed vigorously and incubated in the dark at room temperature for 30 min. The absorbance was measured at 517 nm by an ELISA reader (BioTek, USA), and the percentage of scavenging activity (S_DPPH_) was calculated by the following formula:

S_DPPH_ [%] = 100 × [(A_blank_–A_sample_)/A_blank_]

 A_ blank_ and A_ sample_ indicate the absorbance of the control reaction, and the test compound, respectively. The extract concentration, providing 50% of DPPH- radical scavenging activity (IC_50_), was calculated from a graph, plotted for S_DPPH _against different concentrations of the extract. All assays were performed in triplicates.

**Evaluation of the antioxidant potential of the extract by the Ferric Reducing Antioxidant Power (FRAP) assay**

Different concentrations of *T. persicum* methanolic extract (25, 75, and 125 µg/ml) were added to 2 ml of distilled water and 3 ml of the FRAP reagent, vortexed vigorously, and incubated for 10 min at 37°C. The absorbance of the samples was then measured at 593 nm by an ELISA reader (BioTek, USA). Quercetin and aqueous solution of FeSO_4_ were used as positive control and standard, respectively. All assays were accomplished in triplicates.

**Cell lines and cell culture**

A-375 and Human embryonic kidney 293 (HEK-293) cells were purchased from the National Cell Bank of Iran (NCBI, Pasteur Institute, Tehran, Iran). The cells were cultured in RPMI-1640 medium supplemented with 10% fetal bovine serum (FBS) (Sigma) and 1% penicillin/streptomycin (Sigma) and incubated in a 5% CO_2_ humidified incubator at 37°C. In this study, we used the HEK-293 cells as normal cells to compare the cytotoxicity potential of the extract. 

**MTT assay**

In a 96-well plate, A-375 cells were seeded at a density of 4×10^3 ^cells/well. Cells were allowed to grow to 70% confluency and then were treated with 5, 10, 25, 50, 75, 100, 125, 150, 175, and 200 μg/ml of *T. persicum* methanolic extract for 48 hr. The culture medium was replaced with 100 µl of MTT solution (3- [4, 5-dimethylthiazol-2-yl]-2,5-diphenyl tetrazolium bromide) (5 mg/ml in Phosphate*-*buffered saline (PBS) for three hours, following by replacing with 100 μl of pure Dimethyl sulfoxide (DMSO) to each well. The absorbance was measured at 590 nm using an ELISA reader (BioTek, USA). All assays were performed in triplicates.

**DNA fragmentation assay**

In a 6-well plate, A-375 cells were cultured at a density of 6×10^3 ^cells/well. The cells were then treated with 2, 5, 7, 10, 13, and 15 μg/ml of the extract for 48 hr. After harvesting and collecting cells with 5% Ethylene diamine tetra acetic acid (EDTA)/Trypsin, 400 µl of cell lysis buffer (0.01 M Tris-HCl, pH 8.0, 0.1 M NaCl, 0.025 M EDTA, pH 8, 1% Sodium dodecyl sulfate (SDS) (w/v)) and 0.3 mg/ml of proteinase K were added to pellets and then, incubated overnight at 50°C. Then, 10 µg/ml of RNase was added to the lysate and incubated for 1 hr at 37°C. After centrifugation at 13200 rpm, the supernatant was extracted by using phenol/chloroform/isoamyl alcohol (25:24:1). DNA in the supernatant was precipitated using ethanol for 2 hr at -20 ᵒC and centrifuged at 13200 rpm for 3 min. The dry pellet was dissolved in 15 µl of sterile water, loaded in 1% agarose gel and electrophoresed for one hour. 

**Acridine orange/ethidium bromide (AO/EtBr) nuclear staining**

A-375 cells were seeded on gelatin-coated coverslips in 6-well plates for 48 hr. After that, cells were treated with 5, 10, 13, and 15 µg/ml of the extract for 48 hr. The cells were rinsed with PBS and fixed with 50:50 v/v of cold acetone-methanol solution for 15 min at -20°C, and then, rinsed with PBS twice. A solution containing 100 µg/ml of AO and 100 µg/ml of EtBr (Sigma) was added. After 30 min of incubation, the coverslips were washed with PBS and air-dried and then viewed under a fluorescence microscope (Nikon, Japan).

**Clonogenic assay**

A-375 cells were seeded at a density of 2×10^2^ cells/well in a 24-well plate for 48 hr to attach to the dishes. The cells were treated with 5, 10, 13, 15, and 20 µg/ml of the extract for 7 days. The colonies were fixed with cold methanol-acetic acid (7:1) for 15 min and then, stained with crystal violet (0.5%) for 2 hr. Afterward, the stain was removed, and samples were rinsed with tap water, and allowed to air-dry. Finally, colonies were checked by a stereomicroscope (Olympus, Japan). All assays were performed in triplicates.

**Micronucleus assay**

In a 24-well plate, A-375 cells were seeded at a density of 1.2×10^4 ^cells/well for 48 hr, then, treated with 2, 5, 7, 10, 13, 15, and 20 µg/ml of the extract. After 15 hr, 4 µg/ml of cytochalasin B was added to each well for 28 hr. The cells were then placed in a cold hypotonic solution containing 0.57% KCl and fixed with cold methanol-glacial acetic acid (6:1) mixture. After 48 hr, cells were stained with a solution of 15 % Giemsa (Merck, Germany) in a PBS buffer (pH 7.4) for 15 min and allowed to air-dry. The cells were then viewed and scored by light microscopy at 400× magnification. All assays were performed in triplicates.

***In vitro*** **wound-healing assay**

A-375 cells were plated at a density of 6×10^3 ^cells/well in a 6-well plate and grown to confluence. Next, by using a sterile plastic yellow tip, a scratch was created in the monolayer. To remove damaged or floating cells, the medium was then removed, and the cells were washed with PBS. The cells were treated with 2, 4, 6, 8, and 10 µg/ml of the extract for 48 hr. The plates were then examined by light microscopy for their migration ability. All assays were performed in triplicates.

**Detection of caspases 3/7 activity**

The caspase-3/7 activity was determined using a caspase assay kit according to the instructions of the manufacturer (Kiazist, Iran). A-375 cells were seeded at a density of 4×10^4^ cells/well in a 6-well plate, grown to 70% confluency, and treated with 7, 10, 13, 15, and 17 µg/ml of the methanolic extract of *T. persicum *for 48 hr. The cells were harvested and centrifuged at 1500 rpm for 3 min. The supernatant was discarded, cell lysis buffer, Dithiothreitol (DTT), caspase buffer, and caspase substrate were added to each well and incubated for 2 hr at 37ᵒC. Finally, the light absorbency was measured at 405 nm by an ELISA reader. All assays were performed in triplicates.

**Statistical analyzes**

The data are presented as means ± standard deviations from the three replicates. Statistical analyses were conducted using SPSS (IBM Statistics 25.0). A p-value <0.05 was considered statistically significant. The IC_50_ value was calculated by Excel (Office 2016) and GraphPad Prism software version 8 (California, USA). The area of wound surfaces was measured by using *ImageJ* software ver. 1.40g (NIH, Bethesda, MD, USA).

## Results

**Total phenolic and flavonoid contents of** ***T. persicum*** **methanolic extract**

The average total phenolic content of *T. persicum* methanolic extract, determined by the Folin-Ciocalteu method, was 6.97±0.011 mg GAE/g of dried extract (p<0.05), based on the corresponding standard curve (y=0.015x+0.0753, r^2^=0.994). The average total flavonoid content of *T. persicum* methanolic extract was 46.83 ± 0.0019 mg EQ/g of dried extract (p<0.05), based on the corresponding standard curve (y= 0.0012x+0.0038, r^2^=0.9996).

**Radical scavenging activity of** ***T. persicum* methanolic extract**

To evaluate the free radical scavenging activity of *T. persicum*, DPPH was used as a stable free radical and ascorbic acid as a standard. In the DPPH free radical scavenging assay, 50% inhibition was observed at a concentration of 10±0.002 μg/ml of *T. persicum* methanolic extract, while the IC_50_ value of ascorbic acid was 5±0.005 μg/ml (p<0.05).

**Antioxidant potential of** ***T. persicum* methanolic extract**

The antioxidant potential of *T. persicum* extract was evaluated using the FRAP assay. The average ferric reducing antioxidant power for *T. persicum* methanolic extract and quercetin was 100.25±0.003 and 81.08±0.01 µM of Fe^2+^, respectively (p<0.05).

**Effect of** ***T. persicum*** **extract on the viability of A-375 cells**

**Figure 1:**
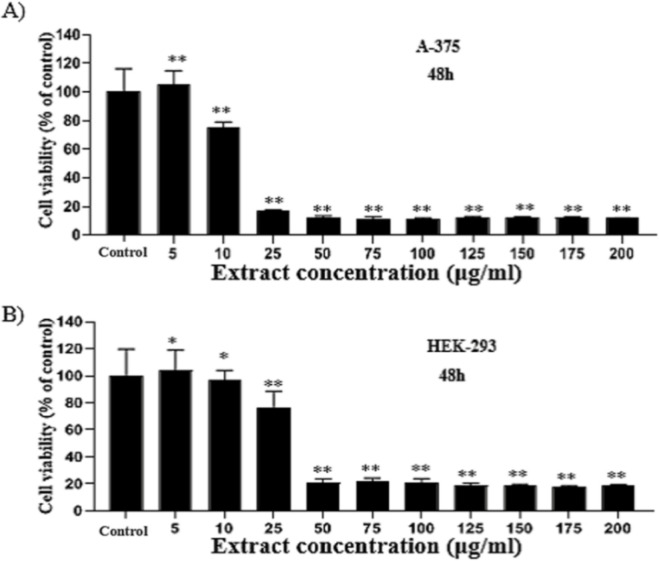
*T. persicum* methanolic extract reduced the cell viability. A-375 (A) and HEK-293 cells (B) were treated with mentioned concentrations of the extract for 48 hr. Each bar shows the mean±SD of three independent experiments (*p<0.05 and **p<0.01 *vs.* control).

A-375 cells were treated with 5, 10, 25, 50, 75, 100, 125, 150, 175, and 200 μg/ml of the extract. The IC_50_ value of *T. persicum *extract for A-375 cells was 13 μg/ml for 48 hr (Figure 1). Treatment of A-375 cells with 5 μg/ml of the extract led to an insignificant increase in the population of viable cells (less than 10%), but higher concentrations reduced the cell viability in a dose-dependent manner (p<0.01) (Figure 1A). Due to the lack of access to the normal human skin cell line, we used the HEK-293 cell line as a replacement. The IC_50 _value of the extract for HEK-293 cells was 49.94 μg/ml (p<0.01) (Figure 1B). 

**Effect of** ***T. persicum*** **extract on the genomic DNA of A-375 cells**

**Figure 2:**
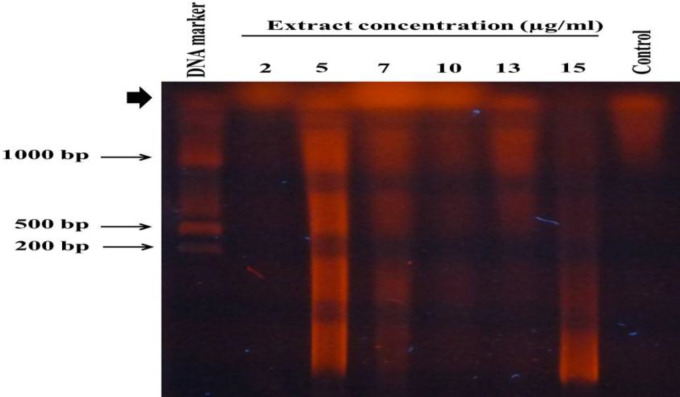
*T. persicum* methanolic extract promoted DNA degradation in A-375 cells. A-375 cells were treated with 2, 5, 7, 10, 13, and 15 μg/ml of *T. persicum* methanolic extract for 48 hr. The genomic DNA was extracted and electrophoresed on a 1% agarose gel and this was followed by ethidium bromide staining. The thick arrow on the top right side of the gel indicates the main band of genomic DNA. The control lane shows non-treated cells

A-375 cells were treated with 2, 5, 7, 10, 13, and 15 μg/ml of the extract for 48 hr, and then, genomic DNA was extracted. Electrophoresis of genomic DNA extracted from each sample in a 1% agarose gel was performed and it was followed by staining with the ethidium bromide. A typical DNA ladder was never observed, while DNA degradation always appeared as a smear in the gel. The smear disappeared in cells treated with higher concentrations including 10, 13, and 15 µg/ml, due to a considerable decrease in the number of viable cells (Figure 2). 

**Effect of methanolic extract of** ***T. persicum*** **on the nuclear status of A-375 cells**

**Figure 3:**
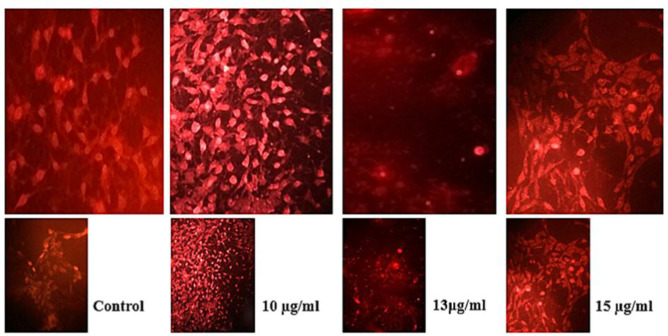
Methanolic extract of *T. persicum* induced nuclear condensation in A-375 cells. The cells showed nuclear condensation and fragmentation. No such changes were observed in non-treated (control) cells

A-375 cells that were treated with sub-lethal or lethal concentrations of the extract were stained with Acridine orange/Ethidium bromide solution. No significant nuclear condensation was detected in control cells, but the cells incubated with concentrations near to or above the IC_50_ (10, 13, and 15 µg/ml of the methanolic extract) for 48 hr showed nuclear condensation (Figure 3). 

**The extract inhibits the clonogenic potentials of A-375 cells**

**Figure 4:**
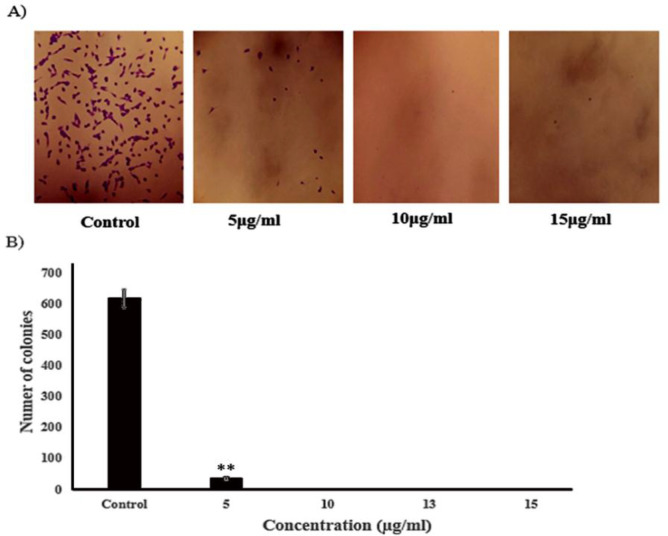
The methanolic extract of *T. persicum* suppressed the colony formation of A-375 cells in a concentration-dependent manner (A). The bars show the mean±SD of three separate experiments. Each treatment was repeated in 10 wells (*p<0.05 versus control) (B)

Figure 4 exhibits the clonogenic survival of A-375 cells treated with 5, 10, 13, 15 µg/ml of the methanolic extract. A reduction in cell division was observed in cells treated with 5 µg/ml of the extract, and 10, 13, and 15 µg/ml of the extract led to suppression of the colony-forming capacity in A-375 treated cells. The plating efficiency (PE) was 57.76% for the control group (non-treated cells), and a surviving fraction (SF) of 1 was calculated for 5 µg/ml of the extract (p<0.01) (Figure 4).

**The** **extract induced micronucleus formation in A-375 cells**

**Figure 5:**
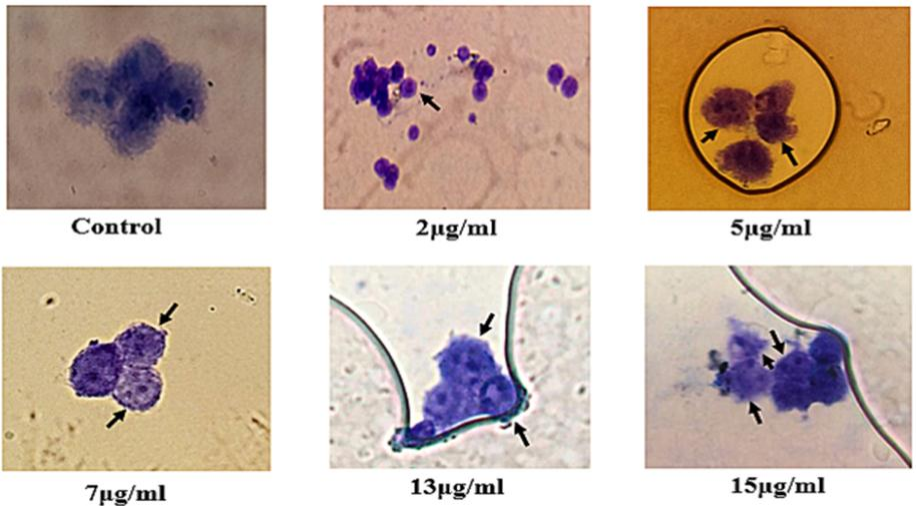
*T. persicum* extract caused micronuclei formation in A-375 cells. Treatment of A-375 cells with sublethal and lethal concentrations of the extract led to a higher frequency of micronuclei or binucleated cells

It is interesting to know that the extract showed genotoxic effects on the cytokinesis of A-375 cells. Our microscopic observations demonstrated that A-375 cells treated with a concentration of 2 µg/ml of the extract were not much different from the control group, but the frequency of binucleated and multinucleated cells or micronuclei in cells treated with 5, 7, 10, 13, and 15 µg/ml of the extract was significantly more than the controls (non-treated) cells (Figure 5). 

***T. persicum*** **extract inhibited the invasion of A-375 cells**

**Figure 6:**
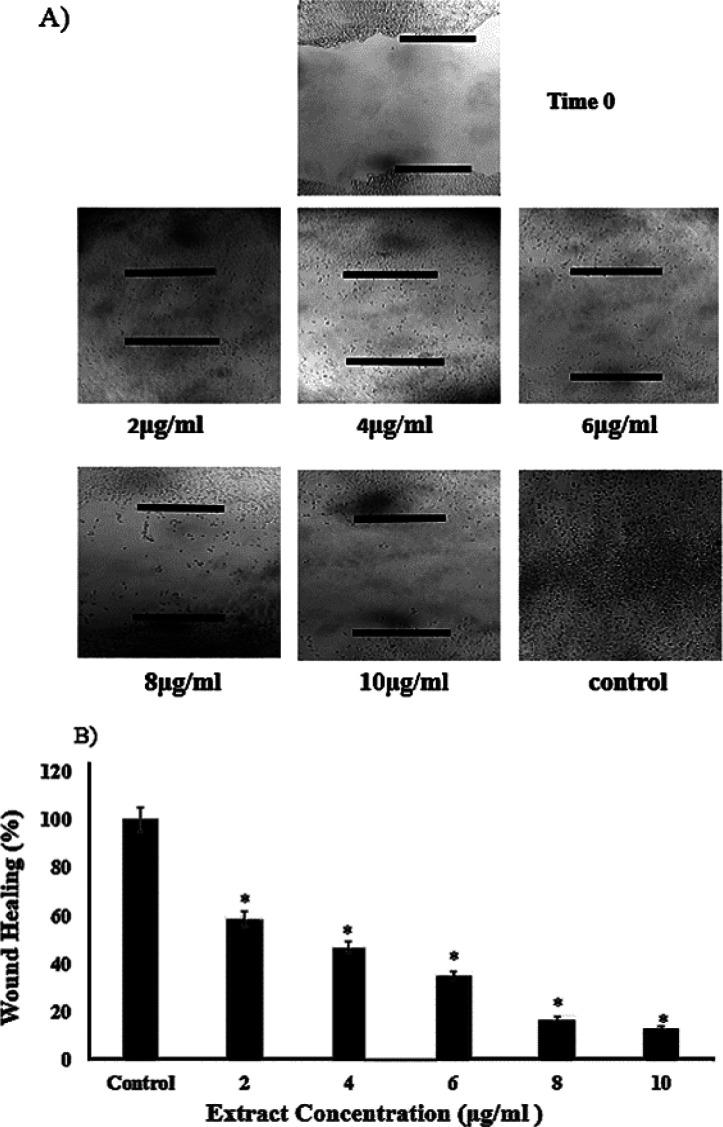
The extract of *T. persicum* inhibited the invasion of A-375 cells. The black short lines represent the border of the wounds (A). The size of wound in cell sheets treated with different concentrations of the extract (B). The bars show the mean±SD of three separate experiments in which each treatment was repeated in 10 wells (*p<0.05 *vs.* control)

To find out whether *T. persicum *extract had any effect on the invasion of A-375 cells, we used the wound-healing (scratch) assay. Figure 6A presents that *T. persicum* extract reduced the proliferation and invasion of A-375 cells in a dose-dependent manner. The wound closure values in cells treated with 0 (non-treated) 2, 4, 6, 8, and 10 μg/ml of the extract were 100, 59, 47.1, 35.4, 17.5, and 13.9%, respectively (p<0.05) (Figure 6B). 

***T. persicum*** **extract induced the activity of caspases 3/7 in A-375 cells**

**Figure 7:**
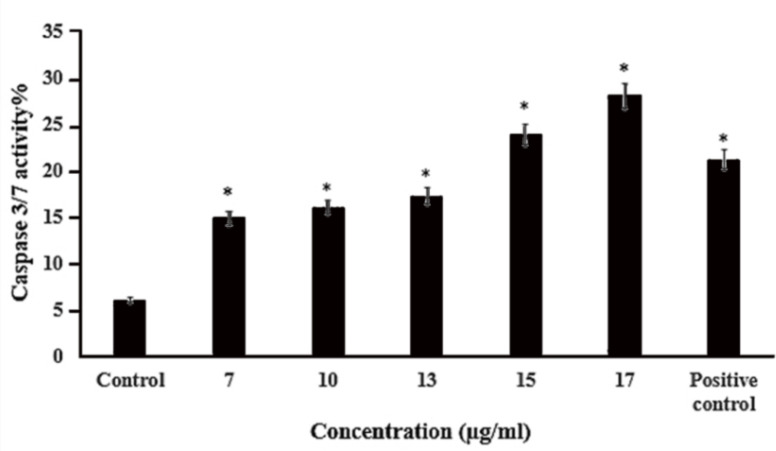
*T. persicum* methanolic extract increased the activity of caspases 3 and 7 in A-375 cells. A-375 cells were incubated at different concentrations of the extract for 48 hr. The positive control contained the substrate of caspases 3 and 7. The bars show the mean±SD of three independent experiments (*p<0.05* vs. *control)

To examine the activity of caspases 3 and 7 levels, A-375 cells were treated with lethal doses of *T. persicum* methanolic extract for 48 hr. As shown in Figure 7, treatment of A-375 cells with concentrations of 13, 15, and 17 μg/ml of the extract in comparison with untreated cells significantly induced the activity of caspases 3 and 7 in a concentration-dependent manner (p<0.05). 

## Discussion

In this study, we aimed to investigate the antioxidant and cytotoxic properties of *T.* *persicum*, as an Iranian endemic species. Our biochemical analyses showed that *T. persicum* methanolic extract contains significant total phenolic and flavonoid contents that are responsible for its remarkable antioxidant and radical scavenging properties. We found that 10±0.002 μg/ml of the methanolic extract is required to scavenge 50% of DPPH radicals (compared to 5±0.005 μg/ml of ascorbic acid) (p<0.05). A previous report indicated that *Teucrium* species are rich in phenolic and flavonoid compounds with very strong biological activities (Stankovic et al., 2011 ▶). In a comparative study, it was found that methanolic extract of *T. persicum* has higher phenolic and flavonoid contents and antioxidant activity than chloroform, ethyl acetate, and water extracts (Miri et al., 2012 ▶). In another study, the essential oil of *T. persicum* showed significant free radical scavenging and antioxidant activities (Monsef-Esfahani et al., 2010 ▶). 

The results of MTT experiments showed that *T. persicum* extract significantly reduced the viability of A-375 cells (IC_50_=13 µg/ml, p<0.01), but had a lower cytotoxic potential against HEK-293 cells (IC_50_=49.94 µg/ml, p<0.01) (Figure 2). Previously, we reported that the methanolic extract of *T. persicum* revealed significant cytotoxic activity against some cancer cell lines (Tafrihi et al., 2014 ▶). 

The results of DNA fragmentation assays revealed that the reduction in the viability of A-375 cells is due to apoptosis induction. DNA fragmentation started at the concentration of 5 µg/ml of the extract and continued at higher concentrations. Interestingly, at the concentration of 2 µg/ml of the extract where a slight increase in the cell viability occurred, DNA fragmentation was not observed (Figure 2). 

Caspases 3 and 7 are both activated during apoptosis, irrespective of the death-initiating stimulus (Walsh et al., 2008 ▶). The caspase activating potential of *T. persicum* extract has not been investigated, yet. In this study, we found that treatment of A-375 cells with *T. persicum* methanolic extract led to the induction of caspase 3/7 activity in a dose-dependent manner (starting at 7 µg/ml, p<0.05) (Figure 7). Several studies have shown that plant extracts or plant-derived compounds possess apoptosis induction potential via induction of caspase 3/7, 8, and 9 activities (Das et al., 2012 ▶; Safarzadeh et al., 2014 ▶; Panicker et al., 2019 ▶). AO/EtBr staining results showed that treatment of A-375 cells with concentrations higher than IC_50_ resulted in nuclear condensation that is one of the hallmarks of apoptosis (Figure 3). 

The results of the clonogenic assay showed that *T. persicum* extract significantly reduced proliferation and colony formation capacity of A-375 cells in a concentration-dependent manner (p<0.01) (Figure 4). In a similar study, it has been shown that *T. polium* extract decreased colony formation potential in some cancer cell lines (Nematollahi-Mahani et al., 2007 ▶; Rajabalian, 2008 ▶). Caryophyllene oxide, linalool, *α*-pinene, *α*-cadinene, *1,4*-cadinadiene, and *α*-terpinyl acetate are the main chemical components of *T. persicum* essential oil (Javidnia et al., 2007 ▶; Miri et al., 2012 ▶). According to reports published,* α*-pinene, *β*-caryophyllene, and *β*-caryophyllene-oxide have cytotoxic effects against various cancer cells (Dahham et al., 2015 ▶; Salehi et al., 2019 ▶). Laboratory investigations suggest that there are one or more compounds in essential oils of *Teucrium* species that may work synergistically to inhibit cancer cells (Stankovic et al., 2011 ▶; Al-Hamwi et al., 2021 ▶). The micronucleus technique is a well-known and sensitive method to assess the genotoxic effects and chromosomal damages potentials of various compounds (Fenech, 2000 ▶). Although evidence shows genotoxicity potentials of other *Teucrium* species (Milošević-Djordjević et al., 2013 ▶; Grujičić et al., 2020 ▶), the genotoxic potentials of *T. persicum* have not been investigated yet. The results of our study showed that *T. persicum *extract has significant genotoxic potential and induced micronucleus formation in A-375 cells (Figure 5). Therefore, it is inferred that the inhibitory effect of *T. persicum* extract on the proliferation machinery of A-375 cells may be due to its genotoxic effects.

Cell proliferation and invasion have a major impact on the wound-healing process (Liang et al., 2007 ▶). However, the sub-lethal concentrations (2 and 4 µg/ml) of the extract did not show a remarkable effect on the cell viability, but treatment of A-375 cells with the extract led to a notable decrease in the wound-healing process in the cell sheets (p<0.05) (Figure 6). Analyzing the phytochemical profile of essential oils prepared from *Teucrium* species revealed that the presence of polyphenols including gallic acid, caffeic acid, quercetin, kaempferol, luteolin, apigenin, etc. may speed up the wound-healing process (Meguellati et al., 2019 ▶; Fallah Huseini et al., 2020 ▶; Chabane et al., 2021 ▶). Therefore, further investigations are needed to prove it and then conclude that probably there are one or more compounds in *T. persicum* that can inhibit the invasion of A-375 cells. 

In conclusion, this is a preliminary investigation on the genotoxic and cytotoxic properties of *T. persicum* methanolic extract on A-375 cancer cells. The results of this study indicated that *T. persicum* has significant antioxidant and cytotoxic effects and can be a good candidate for future investigations. Further analyses on the chemical composition of *T. persicum* extract boost certainly the process of studying its anti-cancer potentials as well as investigating the underlying molecular mechanism(s).

## Conflicts of interest

The authors have declared that there is no conflict of interest.
